# Treatment and Resource Utilization of Gaseous Pollutants in Functionalized Ionic Liquids

**DOI:** 10.3390/molecules29143279

**Published:** 2024-07-11

**Authors:** Jiayu Wang, Rui Wang

**Affiliations:** 1 School of Environmental Science and Engineering, Shandong University, Qingdao 266237, China; 2 Shenzhen Research Institute of Shandong University, Shenzhen 518057, China

**Keywords:** functionalized ionic liquids, gaseous pollutants, catalytic reaction, absorption

## Abstract

With the rapid development of science, technology, and the economy of human society, the emission problem of gas pollutants is becoming more and more serious, which brings great pressure to the global ecological environment. At the same time, the natural resources that can be exploited and utilized on Earth are also showing a trend of exhaustion. As an innovative and environmentally friendly material, functionalized ionic liquids (FILs) have shown great application potential in the capture, separation, and resource utilization of gaseous pollutants. In this paper, the synthesis and characterization methods of FILs are introduced, and the application of FILs in the treatment and recycling of gaseous pollutants is discussed. The future development of FILs in this field is also anticipated, which will provide new ideas and methods for the treatment and recycling of gaseous pollutants and promote the process of environmental protection and sustainable development.

## 1. Introduction

With the advancement of human society, the substantial production of gaseous pollutants has exerted significant pressure on the environment. Gaseous pollutants present in the atmosphere primarily encompass carbon dioxide (CO_2_), hydrogen sulfide (H_2_S), sulfur dioxide (SO_2_), nitrogen oxides (NO_x_), ammonia (NH_3_), and volatile organic compounds (VOCs), predominantly stemming from industrial emissions, coal combustion, automobile exhaust, natural emissions, and other sources [1,2,3,4]. Gaseous pollutants emitted into the atmosphere have a significant impact on the environment, leading to various environmental issues such as acid rain, the greenhouse effect, and ozone layer depletion. Furthermore, prolonged exposure to polluted air can elevate the risk of skin diseases, respiratory illnesses, and cardiovascular conditions. Therefore, it is crucial to regulate gaseous pollutants [5,6,7]. Numerous indigenous resources are depleting to the extent that meeting the increasing demand has become challenging. Simultaneously, resource utilization has emerged as an effective solution to these issues, driven by environmental pressures and resource consumption. Recycling involves the direct use of waste as raw materials or the reprocessing of waste, constituting a crucial component of a circular economy [8,9]. The management and reutilization of gas emissions can create a mutually beneficial scenario for both the environment and the economy.

Ionic liquids (ILs) are a type of salt that exists in a molten state and maintains its liquid form at or around room temperature, typically consisting of an anion and a cation. These fluids possess peculiar properties including extremely low saturation vapor pressure, elevated thermal stability, and a wide range of operational liquid temperatures. As a result of these characteristics, ionic liquids have found widespread applications in various fields such as organic synthesis, nanomaterial preparation, inorganic synthesis, cellulose dissolution, and extraction processes [10,11,12,13].

Functionalized ionic liquids (FILs) are a category of ILs containing specific chemical functional groups that confer distinctive physical and chemical properties to the liquids. FILs typically comprise organic cations and inorganic or organic anions, including cations, such as imidazole salts, pyridine salts, quaternary ammonium salts, etc., and anions, such as halogen ions, tetrafluoroborate ions, hexafluorophosphate ions, etc. FILs can be designed to exhibit extremely selective, active, or stable interactions with specific substances through the careful selection of suitable cations and anions as well as the incorporation of specific functional groups. Due to their high level of versatility and controllability, FILs have demonstrated extensive potential applications in chemical reaction catalysis, material synthesis media, electrochemical energy storage, biotechnology and medicine, environmental treatment and remediation, analytical detection and sensing, as well as other emerging fields [14].

In recent years, the utilization of FILs in capturing and separating gaseous pollutants has emerged as a promising approach for resource recycling. It is capable of creating a special microenvironment, thereby enhancing the reaction rate and selectivity of trapping gaseous pollutants and can also serve as a catalyst or catalyst carrier for various organic and inorganic reactions. This paper provides an overview of the application of FILs in gas pollution treatment and resource recycling over the past decade, discussing the advantages and disadvantages of different methods. The future development prospects of FILs are also examined.

## 2. Synthesis and Characterization of FILs

The synthesis methods of FILs encompass direct synthesis, ion exchange, functional group modification, and others. The direct synthesis method typically involves a one-step preparation of an ionic liquid through an acid–base neutralization reaction or a condensation reaction. This approach offers the advantages of simplicity and speed, without requiring complex equipment or tedious reaction steps. Acid–base neutralization reaction, as one of the earliest methods used to synthesize ionic liquids, is characterized by a strong reactivity and steep product yield. Quaternization introduces peculiar physical and chemical properties to ionic liquids by introducing quaternary ammonium cations [15,16,17]. The ion exchange method involves the preparation of FILs using either ion exchange resin or the inherent ion exchange ability of ionic liquids. The key to this method lies in its precise regulation of cationic ions within ionic liquids, catering to specific application requirements. On the other hand, the functional group modification method entails introducing specific functional groups through chemical reactions based on existing ionic liquids, thereby imparting special functions to them. While this method allows for the preparation of FILs with diverse structures and adjustable functions, it may be constrained by the original properties of the ionic liquids.

The characterization of FILs can be additionally studied to investigate their chemical properties and reactivity. Common methods for characterization include nuclear magnetic resonance spectroscopy (NMR), differential scanning calorimetry (DSC), thermogravimetric analysis (TGA), mass spectrometry (MS), and more [15,16]. Specifically, the elemental composition of the compound can be determined through infrared spectroscopy (IR) analysis of C, H, O, and other elements. NMR analysis, especially hydrogen nuclear magnetic resonance (1H-NMR) and carbon nuclear magnetic resonance (13C-NMR) analysis, can determine the intramolecular hydrogen bond network and chemical bond structure [17,18,19]. TGA and DSC are useful for studying thermal stability, phase transition temperatures, and potential decomposition processes [16,20,21]. Viscometers and potentiometers are used for measuring some liquid properties as well as characterizing electrochemical properties.

## 3. Treatment and Recycling of CO_2_

The primary techniques for CO_2_ removal include adsorption [22,23], absorption [24,25], membrane separation [26], and electrochemical methods [27,28]. While the electrochemical method incurs high equipment costs and the membrane separation method has limited selectivity, the absorption method stands out as a more mature and widely utilized technology. The selection of absorbents plays a crucial role in this method. FILs offer an advantage over traditional absorbents by not only capturing CO_2_ but also converting it into a valuable resource. The principal approach for FILs to recycle CO_2_ involves catalyzing the addition reaction of CO_2_ and epoxide to produce carbonic ester compounds, encompassing ring opening, carbon dioxide insertion, and cyclization steps.

### 3.1. Absorption of CO_2_

**Figure 1:**
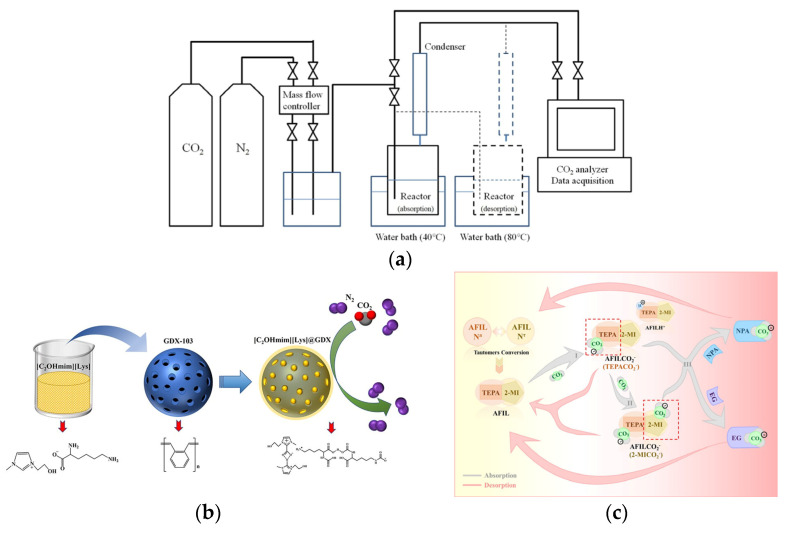
(**a**) CO_2_ absorption process [32]. (**b**) Synthesis of [C_2_OHmim][Lys]@GDX [33]. (**c**) Mechanism of CO_2_ absorption into the [TEPAH][2-MI]/NPA/EG solution [34].

At present, the predominant FILs utilized for CO_2_ absorption are those containing amino functional groups. Zareiekordshouli et al. [29] found that these amino-functionalized ionic liquids (AFILs) exhibit enhanced CO_2_ solubility compared to unfunctionalized counterparts due to their ability to absorb CO_2_ through both physical and chemical processes. The underlying mechanism involves the attack of amino nitrogen atoms on carbon atoms within CO_2_, resulting in the formation of a carbon–nitrogen bond and facilitating CO_2_ absorption [30,31]. And the study conducted by Kang et al. [32] revealed that the manipulation of amino group quantities in the cation has a regulatory effect on the CO_2_ absorption capacity (Table 1). The CO_2_ absorption process is shown in Figure 1a.

**Table 1:** CO_2_ capacity of different ionic liquids.

Ionic Liquids	Temperature K	Pressure Bar	Solubility mol/mol IL	Reference
[Apaeim][OH]	313.15	1	3.75	[32]
[Eaeim][OH]			2.19	
[Paeim][OH]			2.15	
[Apaeim][gly]			4.19	
[Apaeim][ala]			4.72	
[Apaeim][val]			4.44	
[Eaeim][gly]			1.97	
[Eaeim][ala]			2.44	
[Eaeim][val]			2.9	
[Paeim][gly]			2.45	
[Paeim][ala]			2.67	
[Paeim][val]			2.96	
[bmim][ARG]	298	2	0.62	[35]
[bmim][LYS]			0.48	
[bmim][HIS]			0.45	
[bmim][MET]			0.42	
[bmim][LEU]			0.38	
[bmim][GLY]			0.38	
[bmim][VAL]			0.39	
[bmim][ALA]			0.39	
[bmim][PRO]			0.32	
[Bmim][ATZ]	298.15	1	0.14 ± 0.01	[36]
	293.15		0.14 ± 0.01	
	298.15		2.04 ± 0.06	
		2	0.19 ± 0.01	
	323.15	1	0.08 ± 0.03	
		2	0.13 ± 0.02	
[Emim][ATZ]	298.15	1	0.13 ± 0.01	
	293.15		0.13 ± 0.01	
[4NH_2_-BA]	203.15		0.8	[37]
[6NH_2_-NC]			0.78	
[2NH_2_-NC]			0.56	
[Me-Gly]			0.9	
[Ac-Gly]			0.49	
[Ac-PhO]			1.2	

However, AFILs have a high viscosity and increase further during the absorption process, which limits their application in absorption separation. To address this issue, Wu et al. [33] synthesized a novel material [C_2_OHmim][Lys]@GDX (Figure 1b) by immobilizing AFIL [C_2_OHmim][Lys] porous spheres GDX-103. This effectively circumvented the problem of poor mass transfer caused by the elevated viscosity of the ionic liquid. However, the synthesis of the material adds complexity to the experimental procedure. In a separate study, Luo et al. [37] introduced N or O atoms into the AFILs to form intramolecular hydrogen bonds with the aim of reducing viscosity changes during CO_2_ absorption. The formation of intramolecular hydrogen bonds, however, is a time-consuming process, leading to a slow reaction rate in the present experiment. Liu et al. [34] synthesized a novel amino-functionalized ionic liquid [TEPAH][2-MI] and combined it with N-propanol (NPA) and ethylene glycol (EG) to obtain a low-viscosity mixture. The viscosity of the solution before and after CO_2_ absorption was 3.66 mPa·s and 7.65 mPa·s, respectively, significantly lower than that observed when using the ionic liquid alone. The absorption and desorption mechanism of CO_2_ were shown in Figure 1c. However, this approach is hindered by the issue of solvent volatilization, which results in a decrease in absorbent concentration and consequently diminishes absorption efficacy.

Through continuous research, it has been discovered by some researchers that ether-functionalized ionic liquids (EFILs) exhibit higher biodegradability and lower viscosity compared to AFILs [38,39]. Hussain et al. [40] have demonstrated that ether-functionalized pyridine ionic liquids display superior CO_2_ absorption performance in comparison to traditional ILs. Furthermore, increasing the amount of cyanide group in the anion not only enhances the solubility of CO_2_ but also reduces the viscosity and density of the ionic liquid. While the presence of an ether group in the cation has minimal impact on solubility, it significantly improves solubility selectivity. The potential for further research into the absorption of CO_2_ by EFILs is substantial.

Currently, a plethora of novel functional ionic liquids have been employed for the capture of CO_2_. Lv et al. [41] discovered a novel amino acid ionic liquid, [C_2_OHmim][Gly], with the ability to absorb CO_2_. This hydrophilic ionic liquid exhibits high CO_2_ adsorption capacity even after repeated regeneration, although the absorption rate is limited by its physical nature and lack of chemical reaction. In a separate study, Santiago et al. [42] encapsulated an amino acid ionic liquid in a porous carbon capsule (aa-ENIL) for enhanced CO_2_ capture, improving both absorption rate and saturation. However, the packaging process may introduce additional cost and complexity to preparation, while further improvements are needed to ensure long-term stability and regeneration properties for sustained CO_2_ capture performance. In general, the presence of a chemical reaction typically results in a higher absorption rate during the absorption process. However, Zhang et al. [36] have successfully synthesized a series of AFILs capable of ultrafast physical CO_2_ absorption. At atmospheric pressure and room temperature, [bmim][ATZ] exhibits a CO_2_ solubility four times greater than that of [thtdp][Cl], previously recognized as having the highest recorded solubility, thus presenting a novel approach to CO_2_ capture (Table 1).

In general, the current CO_2_ absorption process using FILs still has problems such as limited absorption capacity, increased viscosity, and difficulties in recycling and regeneration. In the future, the field should focus on developing new FILs to improve their efficiency and selectivity in absorbing CO_2_, or on adjusting the structure and composition of ionic liquids to achieve efficient and reversible CO_2_ absorption. Additionally, researchers should explore combining ionic liquids with other technologies, such as membrane separation and electrochemistry, to further enhance the efficiency of separating CO_2_ and reduce energy consumption.

### 3.2. Recycling of CO_2_

**Figure 2:**
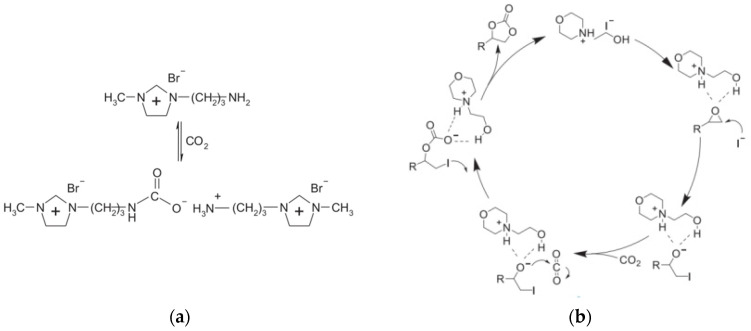
(**a**) AFIL catalyzes CO_2_ conversion to ring carbonates [43]. (**b**) Mechanism for cycloaddition of CO_2_ and epoxide catalyzed by HFIL [46].

The amino groups in the AFILs are a type of basic functional groups, which exhibit good catalytic performance for synthesizing carbonic ester compounds. Additionally, the long N-alkyl chains in the ILs also contribute to improving their catalytic activity [43]. The mechanism of this reaction is illustrated in Figure 2a. Chen et al. [44] employed AFILs as catalysts for the cycloaddition reaction between carbon dioxide and epichlorohydrin, yielding 3-chloro-1,2-propylene carbonate, with a catalytic yield of 85% after a 3-h reaction at atmospheric pressure. Despite the enhanced catalytic effect of AFILs compared to traditional ILs, it has not yet reached optimal performance. To further improve the catalytic capability of AFILs, Liu et al. [45] encapsulated [C_2_NH_2_Mim^+^][Br^−^], an AFIL, within multi-air capsule Zn-ZIF to create a novel material IL@H-Zn/Co-ZIF, which demonstrated a high catalytic yield of CO_2_ addition reaction reaching up to 95%. This approach significantly enhances the catalytic performance of AFILs.

Hydroxyl-functionalized ionic liquids (HFILs) are extensively utilized as catalysts in the synthesis of carbonic ester compounds from CO_2_. The intermolecular hydrogen bond between the epoxide and hydroxyl groups plays a crucial role in promoting epoxide activation, thereby creating a synergistic effect with nucleophilic activation of carbon atoms to polarize C-O bonds (Figure 2b) [46,47]. Yue et al. [48] successfully synthesized [APbim][Lac], with a catalytic yield reaching 97% at 80 °C and 0.5 MPa for 12 h (Table 2). However, the reaction rate is suboptimal due to a high energy barrier for the addition of CO_2_ and epoxide, necessitating a highly active catalyst to overcome this challenge. Wang et al. [49] enhanced the catalyst activity through the design of HFILs containing a higher concentration of active hydrogen atoms to facilitate CO_2_ addition to epoxides. However, the incorporation of functional groups and atoms may lead to a reduction in catalyst stability. The product yield is also influenced by the size of the substituent group on the carbon atom, with an abundance of substituents hindering C-O bond activation [50]. Therefore, it is imperative to explore methods for improving catalytic activity while ensuring the stability of HFILs.

The catalytic mechanism of carboxyl-functionalized ionic liquids (CFILs) is analogous to that of HFILs, with the exception that the hydrogen bond donors are replaced by the carboxyl groups. The experiment by Zhang et al. [53] found that with increased temperature and pressure, the product yield decreased significantly. Therefore, strict control of operating conditions is required for the application of CFILs. The extension of carboxyl-linked alkyl chains can improve the catalytic ability of CFILs. However, synthesizing longer alkyl chains poses considerable challenges. Zhu et al. [52] found that catalytic activity was also improved with ethyl substitution on the N atom nearby. This provides a new way to enhance the catalytic capacity of CFILs. In recent years, the catalytic conversion of CO_2_ by CFILs is relatively rare, and further research is needed to understand its catalytic performance better.

**Figure 3:**
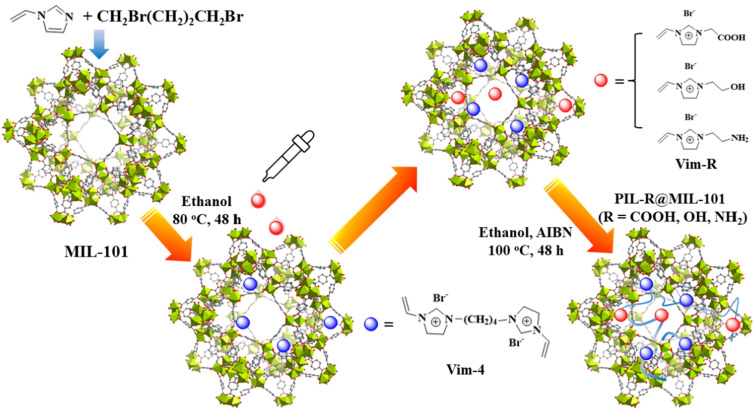
Synthesis of PIL-COOH@MIL-101 [51].

The combination of FILs with other materials represents a promising approach for enhancing catalytic performance. Yao et al. [55] have successfully synthesized a novel material, Co-PMO-IL(X), designed for the efficient conversion of CO_2_ to cyclic carbonates by immobilizing benzotriazole-functionalized ionic liquid onto periodic mesoporous silica-supported cobalt nanoparticles. The catalytic yield of this material ranges from 87.4% to 97.2%, and its activity remains largely unaffected after five cycles. Despite exhibiting favorable catalytic properties in the CO_2_ cycloaddition reaction, its widespread industrial application is hindered by its high cost. Jiang et al. [51] synthesized a series of novel hydrogen bond donor-functionalized poly(ionic liquid)s and @MIL-101 composites for the purpose of CO_2_ capture and catalytic conversion (Figure 3). Notably, PIL-COOH@MIL-101 achieved a high catalytic yield of 92.7% following a 2.5-h reaction at 70 °C and 1.0 MPa (Table 2). This experiment offers the advantage of reducing the use of catalysts and waste generation. However, economic feasibility remains an unavoidable challenge to be addressed in future research endeavors.

Additionally, Shi et al. [56] successfully catalyzed the conversion of CO_2_ to produce quinazoline 2,4(1H,3H)-diones using HFILs. Quinazoline 2,4(1H,3H)-diones exhibit potential anti-cancer, anti-bacterial and anti-mutagenic properties, presenting a novel approach to CO_2_ recycling. However, it is important to note that this experiment was conducted under low concentration conditions of CO_2_ and further investigation is required to determine the catalytic yield under high concentration conditions.

## 4. Treatment and Recycling of H_2_S

In addition to absorption and adsorption methods, H_2_S removal techniques encompass oxidation [57,58], biological processes [59], membrane separation [60,61], and membrane contactor [62,63,64]. Nevertheless, the utilization of these approaches is constrained by issues related to secondary pollution, environmental factors, and membrane fouling. As a novel type of H_2_S absorbent, FILs exhibit a strong affinity for the molecular structure of H_2_S and demonstrate exceptional and efficient absorption capabilities.

### 4.1. Absorption of H_2_S

The primary mechanism for the absorption of H_2_S by AFILs involves the establishment of hydrogen bonds. Zhang et al. [65] investigated the capture mechanism of three triethylenetetramine-functionalized ionic liquids (TETAH-ILs) on H_2_S and determined that the predominant final product consists of a series of hydrogen bond complexes between TETAH-ILs and H_2_S. However, the absorption capacity of H_2_S in this solution was found to be only 0.897–1.018 mol/mol IL (Table 3). In order to enhance the absorption capacity of AFILs, Zhou et al. [66] introduced amino groups on cations, creating H_2_S absorption sites on both cations and achieving an H_2_S absorption capacity of 1.31–1.60 mol/mol IL (Table 3). However, the high viscosity typically associated with AFILs may impact mass transfer efficiency. In response to this challenge, Peng et al. [67] developed a series of supportive proton–ion liquid membranes (SPILMs) that are amino-functionalized and selectively separate H_2_S from CH_4_. The separation performance of the ionic liquid membrane is comparable to that of FILs without viscosity issues. But it should be noted that this method only allows for the isolation of H_2_S and does not facilitate its absorption for recycling purposes. The carboxyl group has weak Lewis base properties, and the tertiary amino group also acts as a Lewis basic group with an isolated electron pair that attracts hydrogen protons in H_2_S [68]. Huang et al. [69] investigated the H_2_S absorption capacity of four tertiary AFILs, and the results showed that [BDMAEE][AcO] had the highest solubility for H_2_S. It was found that the free tertiary amine group in [BDMAEE][AcO] was the most alkaline and therefore better able to absorb H_2_S. Zheng et al. synthesized MLB-PILs, a low viscosity FIL with multiple Lewis bases such as carboxyl groups and tertiary amino groups. The experimental results showed that MLB-PILs had an absorption capacity for H_2_S ranging from 0.65 mol/mol IL to 1.92 mol/mol IL (Table 3). The ionic liquid has low viscosity and high H_2_S absorption capacity. However, compared with anhydrous ILs, MLB-PILs have lower thermal stability and require water to be added during the reaction.

The incorporation of a hydroxyl group is shown to augment the free volume of the ILs, thereby bolstering the solubility of H_2_S [70]. Wei et al. [71] devised a series of weakly basic mercapto carboxylic anion-functionalized ionic liquids (MCA-ILs) for H_2_S absorption. It was observed that while the chemical reactivity between MCA-ILs and H_2_S diminished with decreasing alkalinity following the introduction of a hydroxyl group into the carboxylic acid anion, there was no significant reduction in the solubility of H_2_S in MCA-ILs due to a decrease in absorption enthalpy and enhancement of physical absorption processes. Due to this characteristic, hydroxyl is also utilized in the detection of H_2_S. Huang et al. [72] incorporated monoethanolamine (MEA) into the hydroxyl-functionalized ionic liquid [C_3_OHmim]BF_4_ to enhance the solubility of H_2_S, thereby facilitating the release and oxidation of more HS^−^ ions under suitable potential for detection. This approach circumvents interference from other gases (e.g., CO_2_ and SO_2_) and enables highly sensitive detection of H_2_S. However, the inclusion of MEA will inevitably introduce complexity and escalate the cost associated with the detection process.

**Table 3:** H_2_S capacity of different ionic liquids.

Ionic Liquids	Temperature K	Pressure Bar	Solubility mol/mol IL	Reference
[TETAH][HCOO]-EG	303.15	1	0.897	[65]
[TETAH][BF_4_]-EG			1.018	
[TETAH]Br-EG			1.003	
[DETAH][Im]	298.2	0.1	1.31	[66]
[TETAH][Im]			1.49	
[TEPAH][Im]			1.6	
[BDMAEE][AcO]	298.2		1.044	[69]
			1.004	
			1.073	
			0.95	
[TDMAPAH][Ac]	313.2	1	1.92	[73]
[PMDPTAH][Ac]			1.15	
[TMDAPH][Ac]			0.65	
[P_4444_][MSA]	303.2	1	0.9	[71]
[P_4444_][2-MPA]			0.84	
[N_2224_][2-MPA]			0.75	
[N_2224_][MSA]			0.49	

**Figure 4:**
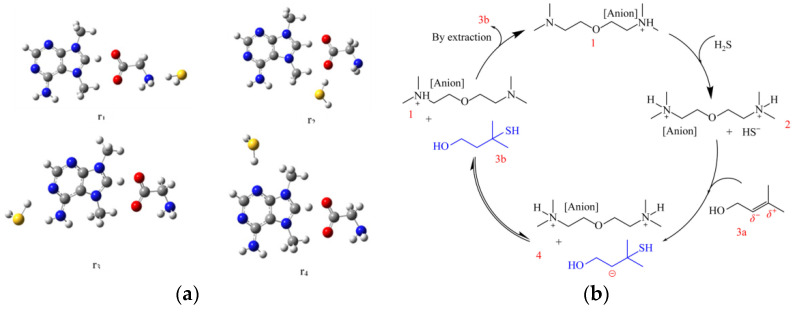
(**a**) Optimal configuration of H_2_S adsorption by [dMA][Gly] [74]. (**b**) Mechanism of H_2_S transformation [75].

Furthermore, novel functional ionic liquids have been utilized for the absorption of H_2_S. Seyedhosseini et al. [74] synthesized ionic liquids containing α-amino acid anions and N7,N9-dimethyladeninium cations and investigated their capacity for H_2_S absorption. The optimal configuration is depicted in Figure 4a. These ILs also exhibit high CO_2_ absorption capabilities, facilitating the capture and separation of CO_2_ and H_2_S. This approach represents a promising new option for the simultaneous recovery of H_2_S and CO_2_.

### 4.2. Recycling of H_2_S

In recent years, there has been a limited number of studies focusing on the direct recycling of H_2_S using functional ionic liquids. Xiong et al. [75,76] have successfully catalyzed the formation of mercaptan alcohols through the addition reaction of H_2_S and epoxide, achieving the recycling of H_2_S using tertiary amine-functionalized protic ionic liquids (PILs). The mechanism for this study is illustrated in Figure 4b. This method eliminates the need for additional solvents to capture and transform H_2_S, exhibits broad applicability, and allows for catalyst reuse, demonstrating promising industrial potential. However, it should be noted that the preparation process for tertiary amine-functionalized ionic liquid is intricate and costly and may also be influenced by water. Therefore, further refinement is necessary to enhance the efficacy of this approach.

At present, the predominant approach for H_2_S recycling involves its oxidation to produce sulfur. Aminuddin et al. [77] demonstrated that metal chloride anion based ionic liquids (MCABILs) achieved H_2_S conversion efficiencies exceeding 90% at a reaction temperature of 100 °C for 6 h. However, it was noted that longer reaction times and reduced performance of MCABILs at lower temperatures were attributed to their higher viscosity. Li et al. [78] synthesized a series of triethylamine hydrochloride·ferric chloride ionic liquids (Et_3_NHCl·FeCl_3_ ILs), which exhibited a sulfur capacity reaching 2.178 wt.% at 303.15 K and 101.3 kPa, with an impressive oxidation efficiency of H_2_S as high as 87.9%. Although the reaction time has been reduced, it still requires 2 h to complete the H_2_S absorption and oxidation process, which may hinder its practical efficiency. The ionic liquid [C_4_mim]_3_PMo_12_O_40_, synthesized in the study conducted by Ma et al. [79], demonstrates high efficiency in H_2_S removal at elevated temperatures. However, its limited solubility in water may compromise the removal efficacy when used in aqueous solutions. Therefore, strict control of reaction conditions is imperative to ensure optimal performance. The introduction of metal groups into ionic liquids has been shown to effectively enhance the oxidation reaction of H_2_S, leading to a significant improvement in the oxidation capacity of ionic liquids for H_2_S. This enhancement ultimately results in an increase in the reaction rate and efficiency, as metal groups typically exhibit excellent redox properties. Therefore, Qiu et al. [80] incorporated a suitable quantity of CuCl_2_ and H_2_O into an iron-based ionic liquid (FE-IL) to establish an Fe/Cu bimetallic catalytic desulfurization system, aiming to address common challenges such as low sulfur capacity, solution degradation, high energy consumption, and poor sulfur quality in aqueous phase oxidation desulfurization. This presents a novel approach for H_2_S oxidation and recycling in the future.

## 5. Treatment and Recycling of SO_2_

SO_2_ is a gas pollutant commonly found in flue gas, and the primary method for its removal is through flue gas desulfurization (FGD). FGD technologies encompass various methods, such as limestone/lime-gypsum, indirect limestone-gypsum, spray drying, ammonia FGD, magnesium oxide FGD, and double alkali FGD [81,82]. While wet desulfurization boasts high efficiency, it often results in the generation of substantial wastewater. Conversely, both dry and semi-dry desulfurization products necessitate further treatment. The use of FILs for SO_2_ capture stands out as an environmentally friendly absorption method due to its minimal production of wastewater or waste.

### 5.1. Absorption of SO_2_

Due to the acidic nature of SO_2_, ionic liquids with alkaline properties are expected to exhibit enhanced absorption capabilities for SO_2_ [83]. The tertiary amino group, known for its Lewis basic functional properties, has been investigated by Geng et al. [84] in their study on the SO_2_ absorption capacity of four tertiary amine-functionalized protic ionic liquids (PILs), which demonstrated absorption capacities ranging from 1.41 mol/mol IL to 1.67 mol/mol IL (Table 4). Extensive research efforts have been dedicated to identifying functional ionic liquids with superior SO_2_ absorption capacity. Cui et al. [85] synthesized a series of halogenated carboxylate ionic liquids with SO_2_ absorption capacities ranging from 3.48 to 4.34 mol/mol IL (Table 4). It has been observed that the absorption capacity of SO_2_ in ionic liquids can be enhanced by incorporating electron-accepting groups such as cyanide group, carboxylic acid group (COOH), or halogen into the anion. The underlying principle of this approach is to increase the electron-withdrawing interaction sites on the anion, thereby reducing the heat of absorption [86,87,88]. However, in 2012, Cui et al. [89] discovered that ionic liquids with ether-functionalized cations could achieve even higher SO_2_ absorption capacities, ranging from 4.43 mol/mol IL to 5.0 mol/mol IL at 20 °C and 1 bar (Table 4). The reaction principle involves the formation of a hydrogen bond between the hydrogen atom in the ether group and the oxygen atom in SO_2_, leading to its absorption through a combination of physical and chemical processes [90]. Due to the strong SO_2_ absorption properties of ether-functionalized ionic liquids, they have been extensively studied by scholars. Zhang et al. [91] observed a competitive interaction between anions and cations in their interaction with SO_2_, consistent with findings by Morganti et al. [92]. Furthermore, researchers noted that the basicity of anions plays a crucial role, as increasing it can enhance the absorption capacity of ionic liquids for SO_2_. However, caution should be exercised as excessively high anion basicity results in greater SO_2_ absorption capacity and more desorption residues [93,94]. Ding et al. [95] synthesized a series of dual-functionalized ionic liquids containing basic anions and ether cations. Among them, [Na(TX-10)][Im] absorbed 6.65 mol/mol IL of SO_2_ at 293.15 K and 1 atm (Table 4), which is significantly higher than traditional ionic liquids. Moisture also affects the SO_2_ absorption capacity. Cui et al. [96,97] investigated acylamido-based anion-functionalized ionic liquids and fluorinated acetylacetonate anion ionic liquids, respectively. Their findings indicated that under 100% humidity, the former exhibited greater SO_2_ absorption capacity compared to dry conditions, while humidity had minimal influence on the latter.

Furthermore, Zhao et al. [98] immobilized the FIL onto porous silica gel as a carrier material and observed that [C_3_O_1_Mim][H_3_CSO_3_]/SiO_2_ exhibited enhanced absorption capacity compared to pure [C_3_O_1_Mim][H_3_CSO_3_]. Gong et al. [99] loaded sulphate FIL onto 2Dh-BN nanosheets, a material known for its ability to capture trace amounts of SO_2_. The combination of functionalized ionic liquids with other materials presents an avenue for improving SO_2_ absorption capacity and offers potential directions for future research.

**Table 4:** SO_2_ capacity of different ionic liquids.

Ionic Liquid	Temperature K	Pressure Bar	Solubility mol/mol IL	Reference
[Na(PEG_400_)][Triz]	303.15	1	0.93	[93]
[Na(PEG_400_)][Bentriz]			0.93	
[Na(PEG_400_)][3-Br-Triz]			0.86	
[Na(PEG_400_)][4-NO_2_-Im]			0.77	
[Na(PEG_400_)][Tetz]			0.66	
[Na(PEG_400_)][SCN]			0.047	
[Na(PEG_400_)][Tetz]			0.66	
[Na(PEG_300_)][Tetz]			0.81	
[Na(mPEG_4_-OH)_2_][Tetz]			0.78	
PEG_400_			0.0078	
[E_3_MIm_2_][Tf_2_N]	323.15	1	1.213	[91]
	293.15		3.307	
[E_1_MIm_2_][Tf_2_N]_2_	313.15		1.291	
[E_2_MIm_2_][Tf_2_N]_2_			1.506	
[E_3_MIm_2_][Tf_2_N]_2_			1.62	
[E_2_Py]Cl^b^	293.15	1	3.924	[100]
[E_3_Mim]Cl^b^			4.367	
[E_3_Py]Cl^b^			4.289	
[E_4_Py]Cl_b_			4.594	
[E_3_Py]Cl_b_	353.15		1.581	
[C_10_Py]Cl			1.334	
[C_3_O_1_Mim][H_3_CSO_3_]	298.15	1	2.621	[98]
[C_5_O_2_Mim][H_3_CSO_3_]			3.106	
[C_7_O_3_Mim][H_3_CSO_3_]			3.453	
[P_4442_][TFSI]	293.15	1	1.43	[97]
[P_4442_][HFA]			2.8	
[P_4442_][BTFA]			4.27	
[P_4442_][TTFA]			4.05	
[MDEAH][MOAc]	313.2	1	1.17	[84]
[MDEAH][EOAc]			1.2	
[BDEAH][MOAc]			1.29	
[BDEAH][EOAc]			1.27	
[P_66614_][6-BrC_5_H_10_COO]	298.15	1	4.34	[85]
[P_66614_][6-ClC_5_H_10_COO]			4.28	
[P_66614_][2-BrC_5_H_10_COO]			3.97	
[P_66614_][C_5_H_11_COO]			3.82	
[P_66614_][BrCH_2_COO]^d^			3.89	
[P_66614_][CH_3_COO]^d^			3.48	
[NEt_2_C_2_Py][SCN]	293.15	1	3.958	[83]
[C_4_CNPy][SCN]			2.917	
[C_4_OPy][SCN]			2.956	
[K(TX-7)][SCN]	293.15	1.01325	3.96	[95]
[Na(TX-7)][SCN]			3.74	
[Li(TX-7)][SCN]			3.51	
[Li(TX-4)][SCN]			2.45	
[Li(TX-10)][SCN]			4.75	
[Na(TX-10)][Tf_2_N]			3.71	
[Na(TX-10)][SCN]			4.89	
[Na(TX-10)][PhO]			5.4	
[Na(TX-10)][Im]			6.65	
TX-4			1.92	
TX-7			3.15	
TX-10			4.52	

### 5.2. Recycling of SO_2_

**Figure 5:**
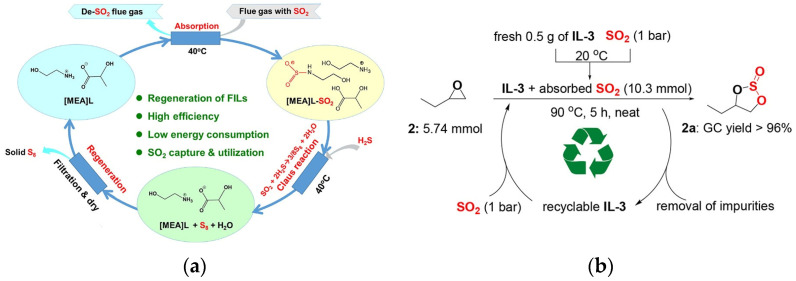
(**a**) The process of SO_2_ regeneration and absorption by Claus reaction in liquid phase [101]. (**b**) Capture and fixation mechanism of SO_2_ [102].

Zhang et al. [101] utilized functionalized ionic liquids for the absorption of SO_2_ and to facilitate the Claus reaction, a widely employed method in desulfurization. The experimental findings demonstrate that, even without the addition of any catalyst and under mild conditions, the reaction between H_2_S and SO_2_ in the absorbent proceeds rapidly and is almost completely converted into solid sulfur (S_8_). The process of SO_2_ regeneration and absorption by Claus reaction in liquid phase is depicted in Figure 5a. Additionally, the reaction effectively regenerates the FILs. However, the high viscosity of functional ionic liquids reduces absorption and regeneration rates, necessitating the addition of glycol during the reaction. Zhao et al. [102] have proposed a novel approach utilizing dual ether-functionalized protic ionic liquids to absorb SO_2_ and directly convert it into cyclic sulfite with a yield of up to 99%. The mechanism of this reaction is depicted in Figure 5b. In this method, the dual ether-functionalized protic ionic liquids serve as both absorber and catalyst, while also being recyclable. Therefore, this method is viable for practical application.

Most of the research in SO_2_ resource utilization focuses on the catalytic oxidation method. In recent years, there has been a greater focus on the research on metal-based ionic liquids as catalysts, while the exploration of FILs as catalysts has received less attention. Therefore, this field needs further development [103].

## 6. Treatment and Recycling of NH_3_

NH_3_ is a gas that can be directly utilized in various industrial applications, such as the production of nitrogen fertilizers and complex fertilizers. The separation technology primarily involves liquid absorption, solid adsorption, membrane separation, and integrated technologies [104]. In recent years, two absorbers have garnered significant attention for NH_3_ absorption: deep eutectic solvents (DES) and FILs [105,106,107,108,109,110]. However, the thermal stability of deep eutectic solvents is generally inferior to that of FILs, thereby limiting their application at high temperatures. The anions and cations of ILs can be tailored to optimize their NH_3_ absorption properties based on the specific application requirements.

Introducing hydroxyl groups into ILs is currently the most widely used method for NH_3_ absorption, as NH_3_ forms strong hydrogen bonds with the hydrogen atoms on the hydroxyl group [111]. Yuan et al. [112] synthesized a series of dual-functionalized ionic liquids (DPILs) by incorporating weak acid properties and hydroxyl functional groups into the ionic liquids. The NH_3_ absorption capacity of these FILs ranges from 2.23 to 3.11 mol/mol IL (Table 5), which is not comparable to traditional water adsorbents. In 2021, Yuan et al. [113] enhanced the experimental methodology and synthesized novel DPILs by incorporating weak acid properties and hydroxyl functional groups into the pyridyl ionic liquid, resulting in an absorption capacity of 1.97–3.43 mol/mol IL for NH_3_ (Table 5). However, these ILs did not exhibit a significant improvement in NH_3_ absorption capacity compared to previous experimental results. Numerous studies have indicated that the solubility of NH_3_ increases with strengthened hydrogen bond interactions. Sun et al. [114] synthesized triazole cation-functionalized ionic liquids (TCFILs) with multiple H-protons in N-heterocyclic cations to enhance NH_3_ absorption capacity. The study revealed that TCFILs exhibited a significantly higher NH_3_ absorption of 4.19–6.17 mol/mol IL at 303.15 K and 1 bar compared to traditional ionic liquids (Table 5), attributed to the acid–base chemical reactions between acidic groups and NH_3_, leading to improved solubility [115]. The study by Shang et al. [116] demonstrated that the 2-methylimidazolium lithium bi (bis (trifluoromethyl sulfonyl) imide) ([2-Mim][Li(NTf_2_)_2_]) has an NH_3_ adsorption capacity of 7.01 mol/mol IL, with one proton site and one Lewis acidic site, as shown in Table 5. This particular ionic liquid not only exhibits higher NH_3_ absorption capacity but also demonstrates superior cycling performance. However, the design and synthesis of such ionic liquids may present technical challenges and may not be suitable for large-scale industrial applications without overcoming difficulties in the synthesis process to ensure stability and reliability.

## 7. Treatment and Recycling of VOCs

The governance of VOCs in the industrial sector can be categorized into two main approaches. The first involves the destruction of VOCs through methods such as incineration, catalytic oxidation, photocatalysis, and biodegradation. However, these methods do not convert VOCs into usable resources [117,118,119,120]. The second approach focuses on the recovery of VOCs using methods such as absorption, adsorption, membrane separation, and condensation. Among these methods, absorption and adsorption have been widely utilized [121,122,123]. In recent years, a significant number of researchers have employed ILs as absorbers for VOCs. Nevertheless, FILs have not been extensively applied in the field of VOC capture compared to common ionic liquids like imidazole [124,125] and pyridine [126,127].

**Figure 6:**
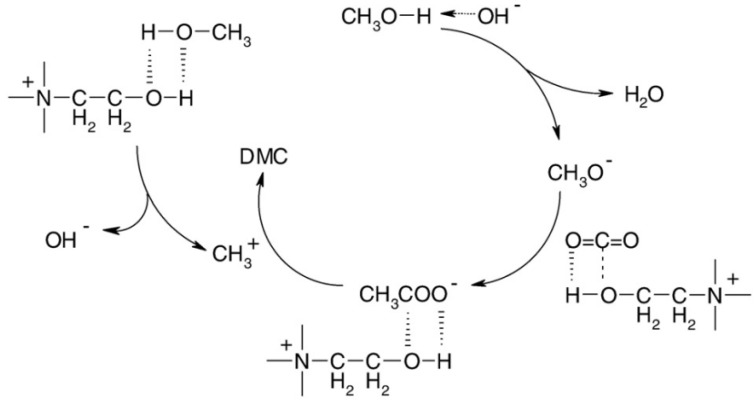
Synthesis mechanism of DMC [129].

The research method for removing VOCs using FILS is analogous to CO_2_ removal, involving the synthesis of ester substances from VOCs for recovery. Miao et al. [128] utilized a long-chain acid-functionalized ionic liquid as a catalyst for alcohol sulfuration at room temperature, resulting in a 99% yield after a 0.5-h reaction. However, the strong acidity of the ionic liquid may induce acid-catalyzed isomerization, polymerization, and other side reactions. Sun et al. [129] and Zhao et al. [130] employed hydroxyl-functionalized ionic liquids (HFILs) as catalysts for the synthesis of dimethyl carbonate (DMC) from CO_2_ and methanol under mild conditions. The mechanism of DMC synthesis is depicted in Figure 6. With the low cost and easy availability of CO_2_, this method not only reduces economic costs for methanol removal but also enables the recovery of both CO_2_ and methanol. This approach shows significant potential for utilization in VOCs and CO_2_ resource management.

Additionally, the majority of ionic liquids are only capable of absorbing either hydrophilic or hydrophobic VOCs. In order to enhance the environmental performance of ionic liquids and achieve simultaneous absorption of both types of VOCs, Fahri et al. [131] synthesized a series of innovative bio-based ionic liquids. Two specific ionic liquids, exylcholinium levulinate and octylcholinium levulinate, demonstrated no saturation at VOC concentrations up to 3000 g/m^3^. Furthermore, their adsorption capacity remained consistent after undergoing five adsorption-desorption cycles. Compared to traditional ionic liquids, these bio-based alternatives offer the advantages of renewability, biodegradability, and low biotoxicity, presenting a new approach for developing environmentally friendly and effective functional ionic liquids.

## 8. Summary and Future Outlook

This paper provides a systematic review of the application of FILs in the field of gas pollution control and resource recycling over the past decade. In summary, FILs exhibit dual functions in treating and recycling gaseous pollutants: they can efficiently absorb gaseous pollutants for recovery and reuse, as well as act as a medium or catalyst for chemical reactions, participating in the process and facilitating the transformation of waste resources. It is noteworthy that during the absorption of gaseous pollutants, functionalized ionic liquids prevent the formation of harmful by-products, aligning with the fundamental principles of green chemistry and demonstrating their environmentally friendly characteristics.

However, despite the significant advantages of FILs in the aforementioned fields, there are still several technical challenges and limitations. The synthesis process of FILs is often intricate, necessitating specific reaction conditions and catalysts, resulting in relatively high production costs and economic pressure for large-scale applications. Additionally, due to the chemical and structural properties of FILs, the regeneration process may not always be efficient. FILs often require regeneration through heating, distillation, or other methods after absorbing pollutants, which can lead to a decline in performance or failure to return to their initial state. This limits their reusability and increases the cost of use. Furthermore, the use of FILs typically requires specialized equipment and process conditions. For instance, precise control of operating temperature, pressure, and other parameters is essential for ensuring the optimal performance of ionic liquids. This requirement leads to increased equipment investment and operational complexity.

Although ionic liquids are considered to be environmentally friendly solvents, their low toxicity is still debated. In some cases, they may pose a potential risk to the environment. For example, ionic liquids may contain certain toxic or harmful components that can negatively impact ecosystems and human health if mishandled or leaked into the environment. In addition, the degradation and recovery of ionic liquids may also produce contaminants that need to be more studied and controlled. Currently, ionic liquids are primarily utilized for laboratory research and specific field applications. Traditional absorbers still dominate in large-scale industrial production. To promote the widespread application of ionic liquid in gas pollutant removal, it is imperative to address the aforementioned key issues and enhance its overall performance.

Looking forward to the future, the application of functional ionic liquids in the field of gas pollution treatment and resource recovery will show the following trends:

(1) Research and development of new functional ionic liquids: By designing more efficient functional groups and optimizing the structure of ionic liquids, it can improve its adsorption capacity, kinetic performance, and selective recognition of gaseous pollutants, so as to achieve a more efficient and highly selective absorption process of gaseous pollutants.
(2) Reduction of the preparation cost: Through process optimization, technological innovation and seeking economical and efficient raw material sources, effectively reduce the preparation cost of functional ionic liquids, so as to promote its popularization and promotion in large-scale industrial applications and achieve a win–win situation of economic and environmental benefits.
(3) Expansion of the application field: The FILs are applied to more types of gas pollutants in the treatment and resource recycling process, in order to give full play to their comprehensive advantages in improving pollution control efficiency, promoting resource recycling and reducing environmental burden, and then enhance their overall benefits and application value in the field of environmental protection.
(4) Development of new resource products: At present, the catalytic conversion of CO_2_ and H_2_S mostly focuses on the synthesis of carbonates and sulfate compounds. In order to expand the application field and meet the diversified industrial needs, further research is needed to explore and develop new high value-added catalytic products, such as fine chemicals containing oxygen or sulfur, functional materials, or new energy materials. The development of these new products will inject new impetus into the development of the environmentally friendly chemical industry.

With the continuous deepening of scientific research, the performance optimization of functional ionic liquids will make remarkable progress, and its application prospects in the treatment of gas pollutants and resource utilization will be more and more broad. Through continuous innovation and technological breakthroughs, we have reason to believe that functional ionic liquids will play an increasingly important role in achieving more efficient environmental protection and resource recycling.

## Figures and Tables

**Table 2:** The catalytic capacity of different catalysts.

Catalyst	Reaction Temperature °C	Initial Pressure MPa	Catalyst Dosage mol%	Reaction Time h	Productivity %	Reference
HEEPzBr	110	1	1	4	91.2	[50]
PIL-COOH@MIL-101	70	1		2.5	92.7	[51]
CMEPzBR	110	2	1	4	99.4	[52]
PS-ImEIMECOOHI_2_	120	2	0.87	2	99.4 ± 1.6	[53]
PAIL-3	60	0.5	5	3	96.1	[54]
[APbim][Lac]	80	0.5	0.5	12	97	[48]

**Table 5:** NH_3_ capacity of different ionic liquids.

Ionic Liquid	Temperature K	Pressure Bar	Solubility mol/mol IL	Reference
[1, 2, 3-TrizH_2_][NO_3_]_2_	303.15	1	4.19	[114]
[1, 2, 4-TrizH_2_][NO_3_]_2_			4.14	
[1, 2, 3-TrizH_2_][CF_3_SO_3_]_2_			6.52	
[1, 2, 4-TrizH_2_][CF_3_SO_3_]_2_			6.17	
[1, 2, 3-TrizH_2_][NO_3_]_2_	313.15		3.64	
[1, 2, 4-TrizH_2_][NO_3_]_2_			3.59	
[1, 2, 3-TrizH_2_][CF_3_SO_3_]_2_			5.69	
[1, 2, 4-TrizH_2_][CF_3_SO_3_]_2_			5.21	
[EtOHim][NTf_2_]	313.15	1	3.11	[112]
[EtOHim][BF_4_]			2.47	
[EtOHim][SCN]			2.23	
LiNTf_2_	313.1	1	3.92	[116]
[Im][NTF_2_]			3.46	
[2-Mim][NTf_2_]			3.04	
[EIM][Li(NTf_2_)_2_]			6.62	
[2-Mim][Li(NTf_2_)_2_]			7.01	
[4-MeOHPy][NTf_2_]	313.15	1	3.43	[113]
[2-EtOHPy][NTf_2_]			3.33	
[2-MeOHPy][NTf_2_]			3.27	
[2-EtOHPy][SCN]			2.33	
[2-EtOHPy][NO_3_]			1.97	
